# Antiproliferative Effects of *Roylea cinerea* (D. Don) Baillon Leaves in Immortalized L6 Rat Skeletal Muscle Cell Line: Role of Reactive Oxygen Species Mediated Pathway

**DOI:** 10.3389/fphar.2020.00322

**Published:** 2020-03-13

**Authors:** Astha Bhatia, Harpal Singh Buttar, Rohit Arora, Balbir Singh, Amritpal Singh, Sarabjit Kaur, Saroj Arora

**Affiliations:** ^1^ Department of Botanical and Environmental Sciences, Guru Nanak Dev University, Amritsar, Punjab, India; ^2^ Department of Pathology and Laboratory Medicine, Faculty of Medicine, University of Ottawa, Ottawa, ON, Canada; ^3^ Department of Biochemistry, Sri Guru Ram Das University of Health Sciences, Amritsar, Punjab, India; ^4^ Department of Pharmaceutical Sciences, Guru Nanak Dev University, Amritsar, Punjab, India

**Keywords:** *Roylea cinerea* (D. Don) Baill., antioxidant, L6, confocal, cell cycle, apoptosis

## Abstract

*Roylea cinerea* (D. Don) Baill. (Lamiaceae) is an indigenous plant of Western Himalayas, and has been used by the native population for the treatment of various diseases such as fever, malaria, diabetes, jaundice, and skin ailments. However, limited proportion of pharmacological and toxicological information is available on the bioactive properties of this plant. Therefore, the present study was designed to explore the anti-oxidant and anti-proliferative activities of *Roylea cinerea*. Methanolic extracts of leaves and stem of *Roylea cinerea* were prepared through maceration procedure and evaluated for the antioxidant activity using hydrogen/electron donating and hydroxyl radical scavenging assay. Significant antioxidant activity was observed for the methanolic extract of leaves in DPPH (EC_50_ 239 µg/ml), molybdate ion reduction assay (29.73 µg ascorbic acid equivalent/mg dry weight of extract) as well as in plasmid nicking assay. Anti-proliferative and apoptotic activity in L6 rat skeletal muscle cell line was done using *in vitro* assays, i.e., MTT, Lactate dehydrogenase, mitochondrial membrane potential assay along with phase contrast, confocal, and scanning electron microscopy. The methanol extract of leaves and stem inhibited the growth of L6 cells with IC_50_ value of 69.41µg/ml and 124.93 µg/ml, respectively, and the lactate dehydrogenase activity was 20.29% and 0.3%, respectively. Cell cycle analysis by flow cytometry exhibited the arrest of cells in G1 and sub-G1 phase by methanolic leaves extract. Furthermore, the results of microscopic and docking analysis strengthened the observation made in the present study regarding the apoptotic mode of cell death in the L6 cell line. The *in vitro* findings of our studies revealed that the bioactive ingredients present in the methanolic extract of leaves and stem of *Roylea cinerea* have the anticancer potential. Further *in vivo* studies are needed to verify the *in vitro* results.

## Introduction

Reactive oxygen species (ROS) are normally produced in the body from the mitochondria and are often termed as ‘redox messengers'. The ROS form an integral part of various intracellular signaling pathways. However, enhanced exposure to xenobiotics and oxidative stress generate prodigious levels of ROS, which in the absence of antioxidant defense pathways can attack cell membrane and alter the structure of cellular macromolecules, protein functioning and may also cause mutations in cellular DNA. Several studies have confirmed the relationship between elevated levels of ROS and carcinogenesis (Weinberg, 1989). The multistep process of carcinogenesis commences through disturbed homeostasis between deviant proto-oncogenes activation and suppression of tumor suppressor genes with critical pathways and biomarkers (Rashid, 2017). Cancer chemoprevention edges on unraveling the potent cost-effective anticancer agents that can specifically influence cellular transformations in the early stages. Despite numerous beneficial effects of synthetic drugs, naturally occurring phytochemicals are preferred as potential anticancer therapies considering the lesser toxicity and fewer side effects.

Naturally occurring phytochemicals have been used in the management of numerous chronic and non-communicable diseases, including cancer and cardiometabolic disorders, and have currently become an important area of research and drug discovery programmes. Basic studies have shown that initiation of cancer is a multistep process that involves tumor initiation, and promotion followed by its progression (Basu, 2018). Extensive efforts are required to unravel the complete mechanism of anti-cancer agents which involves several underlying intracellular signaling cascades. In this context, tailored supplementation of phytochemicals can target these unregulated pathways to inhibit such cellular complications or induce programmed cell death or apoptosis including cyclin dependent kinases and many growth factors. Phytochemicals based anticancer therapies can act as an effective alternative to healthcare costs and side effects in the treatment of cancer with synthetic drugs with an advantage of being inexpensive and accessible. For example, phytochemicals may prevent the carcinogenic effect by capturing the free radicals, and by detoxifying the carcinogen and preventing them to reach the target sites. These natural products may also influence tumor suppressor genes and stimulate the innate immune system, including apoptosis, thereby inhibiting the cellular proliferation pathways and activating various targets such as mitogen-activated protein kinases (MAPKs) and ICE/Ced-3 family proteases (caspases, Singh et al., 2016; Chikara et al., 2018).

*Roylea cinerea* (D. Don) Baillon belongs to the family Lamiaceae. It is an indigenous herb, native to India and grows at an altitude of 1200-3700 m in the Western Himalayas and at the foothills of Nepal. This phytomedicinal plant has been used as a febrifuge, tonic for contusions as well as for treating diabetes mellitus, malaria, and skin diseases (Dobhal and Joshi, 1979; Khare, 2007; Rawat and Vashistha, 2013). The petroleum ether and the chloroform extracts from leaves of *Roylea cinerea* (D. Don) Baillon have been reported with antiplasmodial activity (Dua et al., 2011). The branches of the plant are found to be useful in the treatment of jaundice in infants. Its flowers are used in winters for snuffing to cure coughs (Parkash and Aggarwal, 2010). Several phytochemical compounds have been isolated from the aerial parts of the plant such as labdane-diterpenoids: calyenone, epicalyone, calyone, and precalyone, cinereanoid A, cinereanoid B (Prakash et al., 1979; Sharma et al., 2015); moronic acid (Majumder et al., 1979); cinereanoid C, cinereanoid D, β-lactam, flavonoid glycosides: rutin, isoquercetin, nicotiflorin, martynoside, undatuside A and 50-β-D-glucopyranosyloxyjasmonic acid (Sharma et al., 2017); from chloroform fraction: pilloin, 1-methylindole-3-carboxaldehyde, β-sitosterol, and stigmasterol (Sharma et al., 2015). The compound precalyone (a diterpene) isolated from *Roylea calycina* syn *cinerea* (aerial plant parts) showed anticancer activity up to 143% at concentration of 50 mg/kg in P-388 lymphocytic leukemia in mice in a study conducted (Rastogi and Dhawan, 1990; Pundir and Mahindroo, 2019). Moreover, in another study reported, a target oriented binding analysis to active binding site of Hsp90 and Hsp70 protein which showed potential dual binding affinity of cinereanoid D at 0.1 mg/ml and 1 mg/ml concentration respectively to both the proteins (Sharma et al., 2017).

To the best of our knowledge, no detailed study is available regarding aerial parts of *Roylea cinerea* (D. Don) Baillon with anticancer and antiproliferative potential Thus, keeping in view the scanty literature and some preliminary studies available regarding anticancer potential of *Roylea cinerea* (D. Don) Baillon, the present study was conducted with an objective of unraveling the anticancer potential of methanolic extracts of *Roylea cinerea* (leaves and stem). Further, the mechanistic study was carried out to confirm antiproliferative and apoptotic activity of the methanolic leaves extract of *R. cinerea* in immortalized L6 rat skeletal muscle cell line *via in vitro* assays and microscopic analysis combined with docking analysis of phytoconstituents present in it with PI3K and antiapoptotic proteins (Bcl-2, Bcl-X_L_).

## Methodology

### Plant Collection and Extraction

The plant material (leaves and stem) was collected from District Palampur, Himachal Pradesh, India during the month of May, 2016. The collected plant material was identified and submitted as a voucher specimen for authenticity in the Herbarium, Department of Botanical and Environmental Sciences, Amritsar, India (Accession no. 7376). The plant material (leaves and stem) was completely air dried, coarsely powdered and subjected to maceration procedure in methanol for 2–3 days with agitation at intervals. Literature studies support the use of alcoholic extracts for the extraction of secondary metabolites mainly for polyphenols from plant material as compared to water extracts due to higher extractive potential (Singh et al., 2019). After maceration, the methanolic extracts were concentrated using rotary evaporator (IKA^®^ RV 10) followed by air-drying and stored at −20°C until use.

### Estimation of Total Phenolic Content

Total Phenolic Content was determined using Folin Ciocalteu method given by Yu et al. (2002) with slight modifications. The standard curve for gallic acid (12.5–1,600 µg/ml) was used to calculate the content and expressed as µg gallic acid equivalent/mg dry weight of the extract.

### Estimation of Total Flavonoid Content

Total Flavonoid Content was determined using the method given by Kim et al. (2003) with slight modifications. The standard curve for rutin (12.5–1,600 µg/ml) was used to calculate the content and expressed as µg rutin equivalent/mg dry weight of the extract.

### Antioxidant Activity

#### Hydrogen Donating Activity

Hydrogen donating activity was assessed *via* DPPH (2,2-diphenyl-1-picrylhydrazyl) free radical scavenging assay. The assay was performed as per the method given by Blois (1958) with minor modifications. The plant extracts (30 µl) with varying concentrations (25–1,000 µg/ml) was incubated with 200 µl of DPPH dissolved in methanol for 30 min at 37˚C. Following the incubation, the final absorbance was measured using Biotek multi-well plate reader at 517 nm against a blank solution. The % radical scavenging activity was calculated as:

$$\left(OD_{BLANK-}OD_{SAMPLE}\right)/OD_{BLANK}\times 100$$

Gallic acid was used as standard and evaluated at a varying concentration (5–100 µg/ml)

#### Electron Donating Activity

Electron donating activity was assessed *via* molybdate ion reduction assay. Plant extracts were evaluated for their ability to reduce molybdate ion as per the method given by Prieto et al. (1999) with slight modifications. The extract (25 µl) was mixed with 250 µl reagent (0.6 M H_2_SO_4_, 28 mM sodium phosphate, and 4 mM ammonium molybdate). The final reaction mixture (300 µl) was heated at 95˚C for 1.5 h. The absorbance was read at 695 nm using Biotek multi-well plate reader. The antioxidant activity was calculated using a standard curve for ascorbic acid (20–200 µg/ml) and expressed in terms of ascorbic acid equivalents (**Supplementary Figure 6**).

#### Hydroxyl Scavenging Activity

The DNA nicking assay was performed according to Lee et al. (2002) with minor modifications. Fenton reagent (30 mM H_2_O_2_, 80 mM FeCl_3_, 50 mM ascorbic acid) was used as a negative control. The plant extracts concentrations (12.5–400 µg/ml) were mixed with freshly prepared negative control and pBR 322 plasmid DNA. The final reaction mixture was made up to 20 µl using sterile distilled water. Electrophoresis was performed after loading different reaction mixtures into 1% agarose gel at 60 V for 1.5 h. Bands were visualized using the Gel Doc XR system (Bio-Rad, USA) and quantified using Gel Quant and Labimage Platform software (free version) software.

### Antiproliferative Activity

#### MTT Assay

The plant extracts (leaves and stem) from *Roylea cinerea* (D. Don) Baill. were evaluated for their anti-proliferative activity in L6 skeletal muscle cell line using the method given by Liu et al. (2006) with minor modifications. The L6 cells were seeded in 96 well plate with a density of 10x10^3^ cells/well and incubated for 24 h. After incubation, cells were treated with varying concentration of plant extracts (25–1,600 µg/ml) for 24 h at 37°C and 5% CO_2_. Following incubation, MTT (100 µl) was added to each well after carefully removing the media and further incubated for 4 h. Post-treatment, the solution was aspirated from each well and insoluble formazan was dissolved in DMSO (100 µl). The absorbance was recorded at 540 nm using Biotek Synergy HT multi-well plate reader against blank. The percentage inhibition was calculated as:

$$\left(\left(OD_{BLANK-}OD_{SAMPLE}\right)/OD_{BLANK}\times 100\right)$$

Growth inhibitory concentration (the concentration of the sample with 50% death of the cells, i.e., G1_50_)

#### Lactate Dehydrogenase Assay

The cellular damage was assessed *via* the enzyme lactate dehydrogenase which is present in all cells and released rapidly during cell damage. The assay was performed according to the method given by Abe and Matsuki (2000) to evaluate cellular damage or cell death *via* necrosis. The cells were seeded with density 3x10^5^ in 24 well plate at 37˚C and 5% CO_2_ for 24 h. After incubation, the cells were treated with GI_50_ and GI_70_ of plant extracts calculated from MTT assay for 24 h. Post-treatment, the supernatant (100 µl) was collected and transferred to 96 well plate followed by the addition of 100 µl LDH buffer (2.5 mg Lithium lactate, 2.5 mg NAD^+^, Tris-HCl (pH 8.2) dissolved in 0.1% Triton-X; 100 µl MTT and 1 µl methoxyphenazine methosulfate). The reaction mixture was incubated for 30 min in dark followed by the addition of stop solution (100 µl) 1M acetic acid. Absorbance was read at 570 nm using Biotek Synergy HT multi-well plate reader against blank and % enzyme activity was calculated as:

$$\left(\left(OD_{BLANK-}OD_{SAMPLE}\right)/OD_{BLANK}\times 100\right)$$

#### Assesment of Cell Morphology Through Microscopic Studies

The morphological features of normal and apoptotic cells were examined through phase-contrast microscope as per the method given by Ramasamy et al. (2013). Nuclear morphology was analyzed by confocal microscopy using DAPI and Ethidium Bromide-acridine orange (EB/AO) to detect apoptotic cells (Kasibhatla et al., 2006). The cells were analyzed using the Nikon A1R Laser Scanning Confocal Microscope (Nikon Corporation, Japan) with NIS-Elements AR analysis software (version 4.11.00). Scanning electron microscopy was performed as per the method given by Ye et al. (2012) to study the surface morphology of normal and apoptotic cells using scanning electron microscope (Carl Zeiss SUPRA55).

#### Intracellular Reactive Oxygen Species Content

ROS levels were determined in L6 rat skeletal muscle cells using DCFH-DA probe (Dichloro-dihydro-fluorescein diacetate) as per the method given by Deng et al., 2013 with slight modifications. Cells were cultured in a 6-well plate with density 5x10^5^ cells/well (2 ml) and incubated for 24 h. The cells were treated with IC_30_, IC_50_, and IC_70_ of *Roylea cinerea* (D. Don) Baill. methanolic leaves and stem extract for another 24 h. After treatment, cells were incubated for 30 min with the DCFH-DA probe (10 µg/ml) at 37°C in the CO_2_ incubator. Following incubation, the cells were harvested and washed twice with 1x PBS (1 ml) and immediately observed for oxidative burst with Biotek multi-well plate reader for fluorescent intensity (485 nm excitation and 528 nm emission) as well as BD Accuri TM C6 Flow Cytometer (excitation 488 nm, emission 535 nm, FL-1 channel, events recorded 10,000 per sample), and the results obtained were expressed in terms of % intracellular ROS in cells.

#### Measurement of Mitochondrial Membrane Potential

MMP was determined in L6 rat skeletal muscle cells using Rhodamine-123 as per the method given by Deng et al. (2013) with slight modifications. Cells were cultured in a 6-well plate with density 5x10^5^cells/well (2 ml) and incubated for 24 h. The cells were treated for 24 h with IC_30_, IC_50_and IC_70_ of *Roylea cinerea* (D. Don) Baill. methanolic leaves and stem extract and incubated for 30 min with the Rhodamine-123 (10 µg/ml) at 37°C in the CO_2_ incubator. Following incubation, cells were harvested and washed twice with 1x PBS (1 ml) and immediately observed with Biotek multi-well plate reader for fluorescent intensity (485 nm excitation and 528 nm emission) as well as BD AccuriTM C6 Flow Cytometer (excitation 511 nm, emission 535 nm, FL-1 channel, events recorded 10,000 per sample).

#### Cell Cycle Analysis

Cell cycle analysis was performed to analyze DNA content in different phases of cell cycle as per the method given by Jordan et al. (1996) with slight modifications. L6 cells plated in six well plate (5x10^5^) were treated with various concentrations of *R. cinerea* leaves extract for 24 h. After treatment, cells were centrifuged to obtain a pellet and washed with chilled 500 µl of PBS. Further, the cells were fixed with 70% ethanol at 15°C for 30 min. After fixation, the cells were again centrifuged to obtain a pellet and washing step was repeated followed by incubation of cells with RNAase (10 µg/ml) and propidium iodide stain (10 µg/ml) for another 30 min. After incubation, cells were analyzed immediately for DNA content using BD AccuriTM C6 Cytometer (excitation 488 nm, emission 600 nm, FL2 channel, events recorded 10,000 per sample). The histogram obtained from cell cycle distribution was analyzed by BD AccuriTM C6 software and expressed in terms of % cells in each phase of cell cycle.

### Molecular Docking Studies

The methanolic extract of leaves of *R. cinerea* exhibited substantial anti-proliferative activity, therefore the docking studies were carried out for the chemical constituents already reported in this plant (Sharma et al., 2015; Sharma et al., 2017). The chemical structure and molecules for which docking was carried out are provided in **Supplementary Figure 1**. Ligand structures were obtained from PubChem (https://pubchem.ncbi.nlm.nih.gov/search/search.cgi) and prepared using chemsketch tool. In this study, the protein structure of the target proteins PI3K (PDB ID: 1E8Z), Bcl2 (PDB ID: 4IEH), Bclxl (PDB ID: 4QNQ) and a binding pockets was obtained from the protein data bank (www.rcsb.org) (**Supplementary Figure 2**). The preparation of the target proteins was done using Swiss PDB viewer v4.1.0 involved energy minimization. Further, polar hydrogen atoms were added to target protein and for the computation of partial atomic charge using AutoDock4. Hetero-atoms present in the protein structures 1E8Z, 4IEH, and 4QNQ were removed prior to autodock analysis. The automated docking of specified ligands into protein binding pocket was done considering Gasteiger charges for each atom present in the target. Three-dimensional affinity grid size for 1E8Z was 51.201, 12.569, 28.184 (x, y, and z), for 4IEH was 14.216, 21.636, 11.709, and for 4QNQ was 52.232, 7.115, -11.211 used on the geometric center of the target protein. Docking algorithm was run using Cygwin software to obtain the binding energy data for each run. Visualization and analysis of the results were done using UCSF chimera 1.11rc.

### Statistical Analysis

The data were analyzed using regression analysis and implemented by best-fit-model. The regression equation obtained was used for the calculation of TPC, TFC, EC_50_, and GI_50_ values. In addition, one-way analysis of variances (ANOVA) and Tukey's test was employed for comparing means of different concentrations of the same extract assuming variances are equal using IBM SPSS version 16.0 software. The difference in % ROS and % depolarized cells between the control cells and treated cells was analyzed by Student's Independent t-test. The results were expressed as mean ± SE.

## Results and Discussion

**Figure 1:**
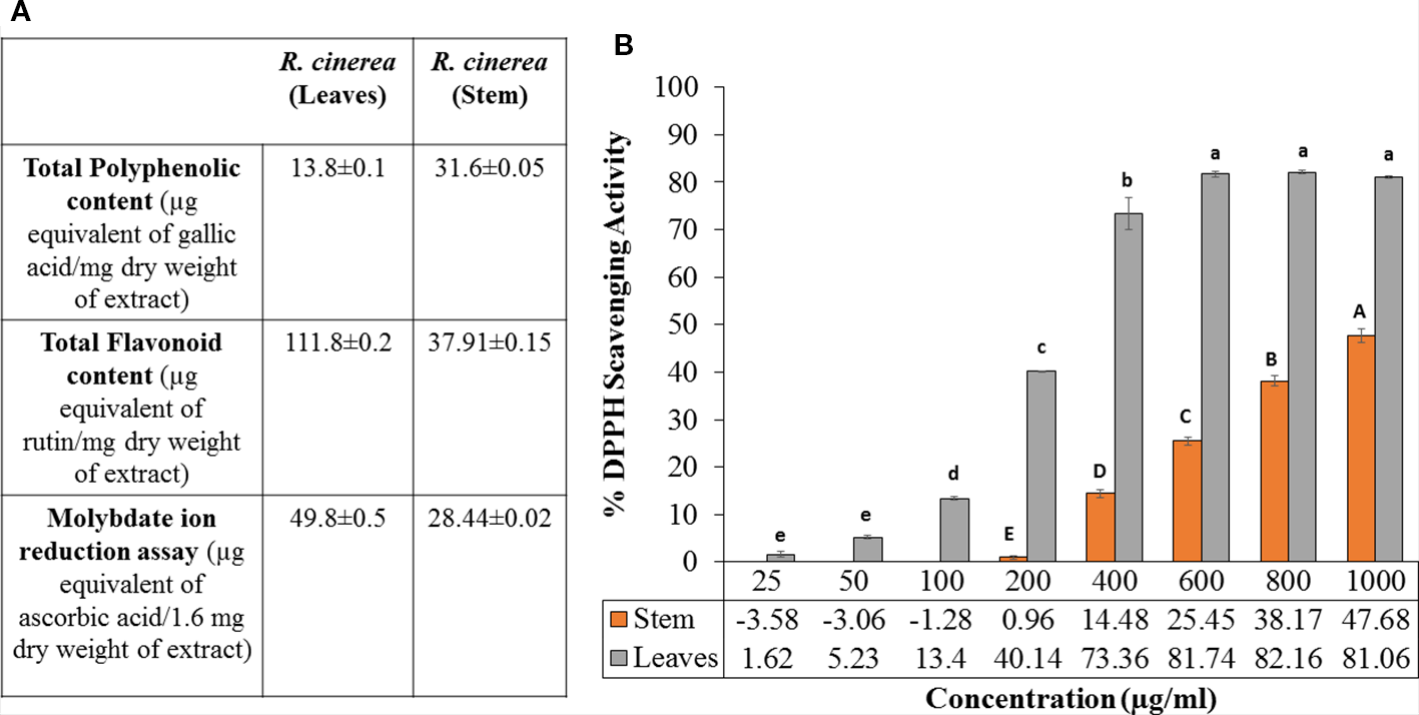
**(A)** Molybdate ion reduction potential, total phenolic content (TPC) and total flavonoid content (TFC) values of leaves and stem extract of *R. cinerea*), **(B)** DPPH radical scavenging potential of leaves and stem extract of *R. cinerea.* (Values are mean ± S.E. of three parallel measurements. Different letters indicate significant differences between different concentrations of *R. cinerea* (leaves and stem) methanolic extracts (p < 0.05, Tukeys HSD test, (F-ratio- 381.283 (*R. cinerea* (stem)), 833.829 (*R. cinerea* (leaves))).

Previous reports have confirmed the relation between the intake of natural phytochemicals and the low incidence of various diseases such as heart diseases, diabetes, cancer and the process of aging. Furthermore, medicinal plants demonstrating higher antioxidant activity have been reported to contain a high amount of phenolic compounds. Thus, such plants can act as a potential source of antioxidants to combat various diseases including cancer. A perusal of literature showed various medicinal plants with high phenolic content associated with their chemopreventive as well as anticancer activity. Such reports include Lichochalcone A (LCA) from licorice, Cinnamtannin B1 from litchi, green tea (*Camellia sinensis*), ethanolic extract of *Tragopogon porrifolius*, blackberry, apples, *Prunus avium* (cherries), *Fagopyrum tataricum*, *Emblica officinalis,* etc (Wang et al., 2008; Serra et al., 2010; Lou et al., 2011; Zheng et al., 2012; Wen et al., 2015; Al-Rimawi et al., 2016; Chen et al., 2017). In the present study, total phenolic content (TPC) for methanolic extracts of leaves and stem of *R. cinerea* was obtained as 13.86 and 31.65 µg GAE/mg dry weight of the extract. The total flavonoid content (TFC) values for both the extracts was obtained as 111.87 and 37.91 µg RE/mg dry weight of the extract respectively (**Figure 1A**). Further, methanolic extracts of leaves and stem of *Roylea cinerea* (D. Don) Baill. were evaluated for their antioxidant potential in terms of their hydrogen donating capacity. Among leaves and stem extract, the former exhibited higher DPPH radical scavenging activity with IC_50_ of 239 µg/ml as compared to the latter exhibiting IC_50_ of 1,076.42 µg/ml (**Figure 1B**). Gallic acid was used as standard and it showed the IC_50_ of 8.56 µg/ml. The extracts were also evaluated for their electron-donating ability *via* molybdate ion reduction assay. The *R. cinerea* leaves extract exhibited 49.84 µg ascorbic acid equivalents/1.6 mg dry weight of extract and stem extract showed comparatively lower activity of 28.44 µg ascorbic acid equivalents/1.6 mg dry weight of extract (Y=0.0074x-0.1115, R^2^ = 0.993)(**Figure 1A**).

**Figure 2:**
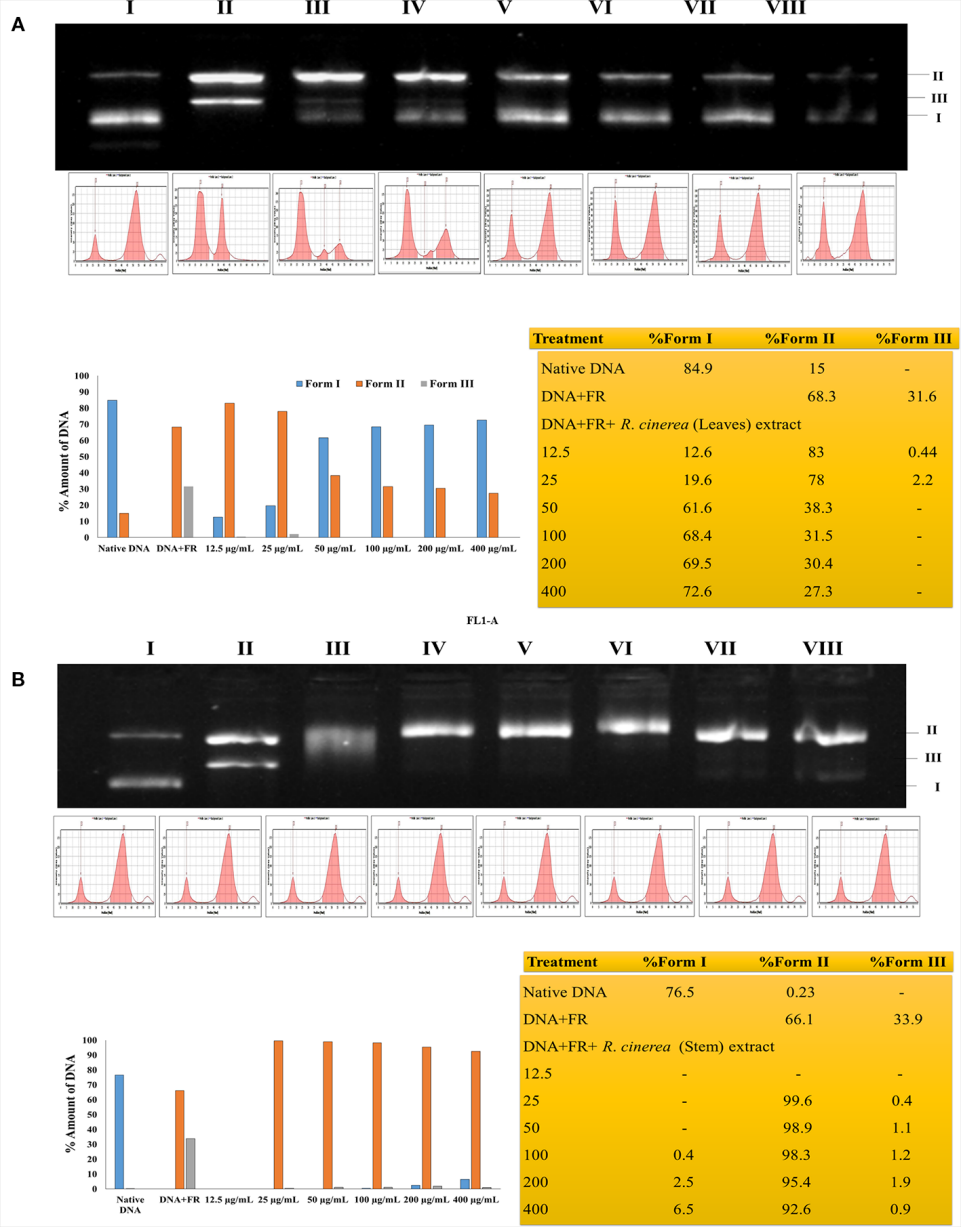
**(A)** Protective effects of leaf extract obtained from *R. cinerea* against Fenton's reagent (FR) induced DNA damage (nicking) in pBR322. Lane I: native DNA, Lane II: DNA+FR, Lane III-VIII: DNA+FR+leaf extract (12.5–400 µg/ml). **Form I**: Supercoiled DNA, **Form II**: Linear (nicked) DNA and **Form III**: Relaxed circular DNA. **(B)** Protective effects of stem extract obtained from *R. cinerea* against Fenton's reagent (FR) induced DNA damage (nicking) in pBR322. Lane I: native DNA, Lane II: DNA+FR, Lane III-VIII: DNA+FR+stem extract (12.5–400 µg/ml). **Form I**: Supercoiled DNA, **Form II**: Linear (nicked) DNA and **Form III**: Relaxed circular DNA.

The TPC and TFC values clearly indicated that the leaves extracts were rich in flavonoid content and the stem extract showed higher content of phenolic compounds. In the previous study, stem and leaves extract of *R. cinerea* were investigated for the presence of seven polyphenols including gallic acid, rutin, catechin, quercetin, umbelliferone, epicatechin, and kaempferol. The extracts showed the presence of high content of rutin leaves as compared to the stem (Bhatia et al., 2019). Rutin and its metabolites contain vicinyl dihydroxy groups which are mainly responsible for its free radical scavenging properties (Yang et al., 2008). The potential of polyphenols is affected by the position as well as the number of hydroxyl groups attached to the aromatic ring combined with their glycosylation or the presence of other hydrogen donating groups such as –SH, -NH (Cai et al., 2004). These functional groups have also been implicated in inhibiting oxidation progression *via* radical chain-breaking properties (Ghasemzadeh and Ghasemzadeh, 2011). The DNA protective ability against the OH radical was assessed through plasmid nicking assay with slight modifications. The leaves extract of *R. cinerea* were able to protect the native DNA (more or less), i.e., supercoiled DNA (form I) in a dose-dependent manner upto 72.6% (**Figure 2A**) as compared to the stem extract with 6.5% of Form I and 92.6% of Form II DNA (linear DNA) (**Figure 2B**). The antioxidant results in the case of leaves extract showed higher TFC content, DPPH radical scavenging activity and molybdate ion reduction ability which can be corroborated with the presence of rutin in high amount in leaves. These findings have been confirmed with the literature survey (Bhatia et al., 2019; Pundir and Mahindroo, 2019). The stem extract showed less antioxidant activity, as well as low TFC value but TPC value was higher as compared to leaf extract.

**Figure 3:**
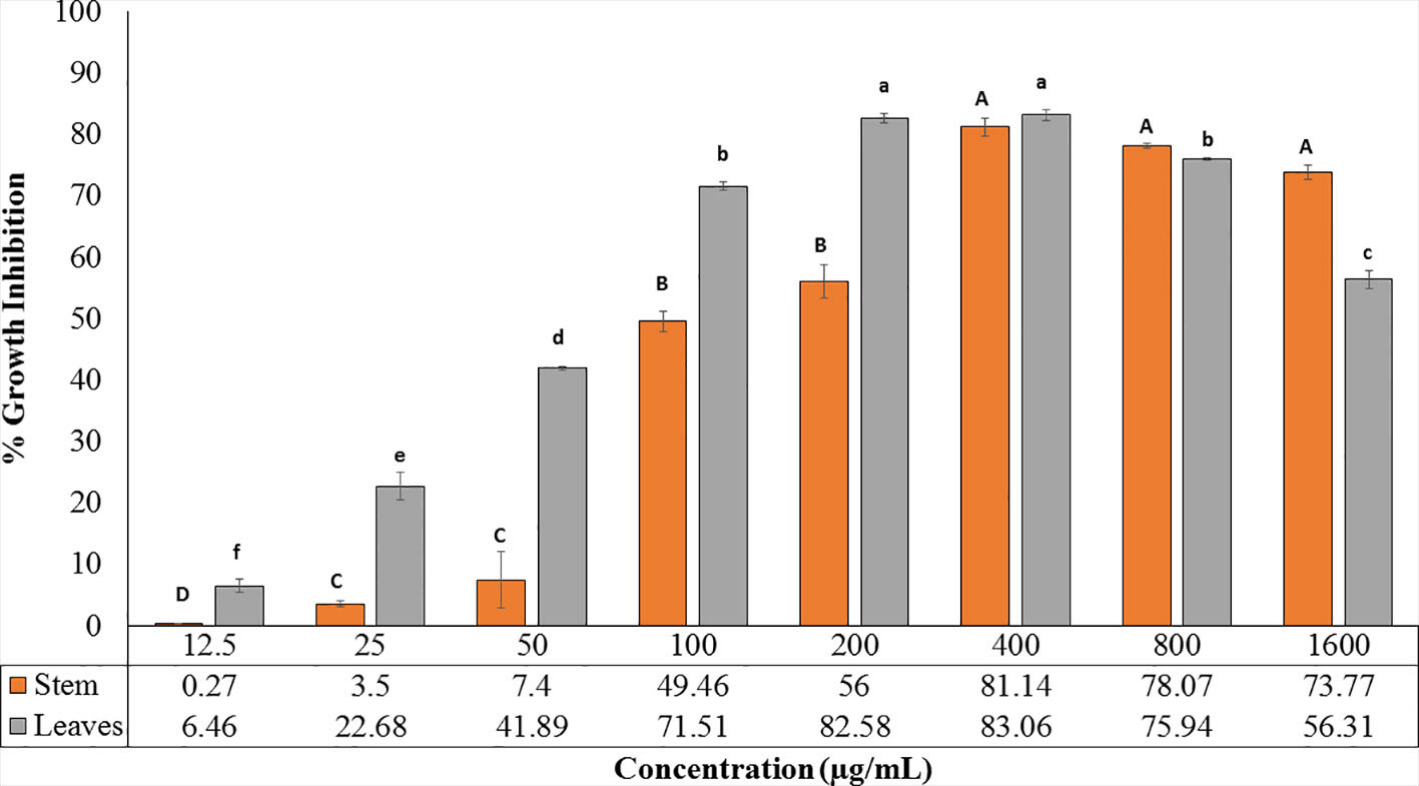
Cytotoxic potential of leaves and stem extract of *R. cinerea* evaluated by MTT assay. (Values are mean ± S.E. of three parallel measurements. Different letters indicate significant differences between different concentrations of *R. cinerea* (leaves and stem) methanolic extracts (p < 0.05, Tukeys HSD test, (F-ratio- 277.429 (*R. cinerea* (stem)), 663.915 (*R. cinerea* (leaves)).

The extracts obtained from *R. cinerea* leaves and stem were also evaluated for anti-proliferative activity in the immortalized L6 skeletal muscle cell line by MTT assay. Both the extracts showed varying degrees of inhibitory potential against cell growth in dose-dependent manner (**Figure 3**). The leaves and stem extract explicited a considerable level of inhibitory potential with 83.06% and 81.14% inhibition at 400 µg/ml with GI_50_ of 69.41 µg/ml and 124.93 µg/ml respectively (**Figure 3**). The antiproliferative potential of both the extracts was substantially corroborated with the antioxidant potential. The majority of the reported anticancer herbal medicines have been proved to be efficient in several clinical reports and experimental research for the prevention as well as treatment of cancer to a better extent (Konkimalla and Efferth, 2008; Reuben et al., 2012). In a previous study, *Roylea cinerea* (D.Don) Baill. showed anticancer activity against SK-Mel 41, U-87 MG, Hela, MDA-MBA-231 cell line with GI_50_ 131.8 µg/ml, 275.4 µg/ml, and 302.0 µg/ml, respectively (Bahuguna et al., 2015). The mode of death (apoptosis) was confirmed by comparing the LDH activity of the treated L6 cells (GI_50_, GI_70_) which showed decreased LDH activity. Cancer cells have been reported with a high glycolysis rate for survival. Instead of entering further into citric acid cycle, pyruvate is converted into lactate *via* lactate dehydrogenase enzyme. This step consumes NADH and produces NAD^+^, consequently inducing a decrease in mitochondrial membrane potential which ultimately causes apoptosis (Franco-Molina et al., 2010). The leaves and stem extract-treated cells caused 20.09% (GI_50_), 39.32% (GI_70_), and 0.3% (GI_50_), 14.49% (GI_70_) LDH activity respectively which confirmed the apoptotic mode of cell death.

**Figure 4:**
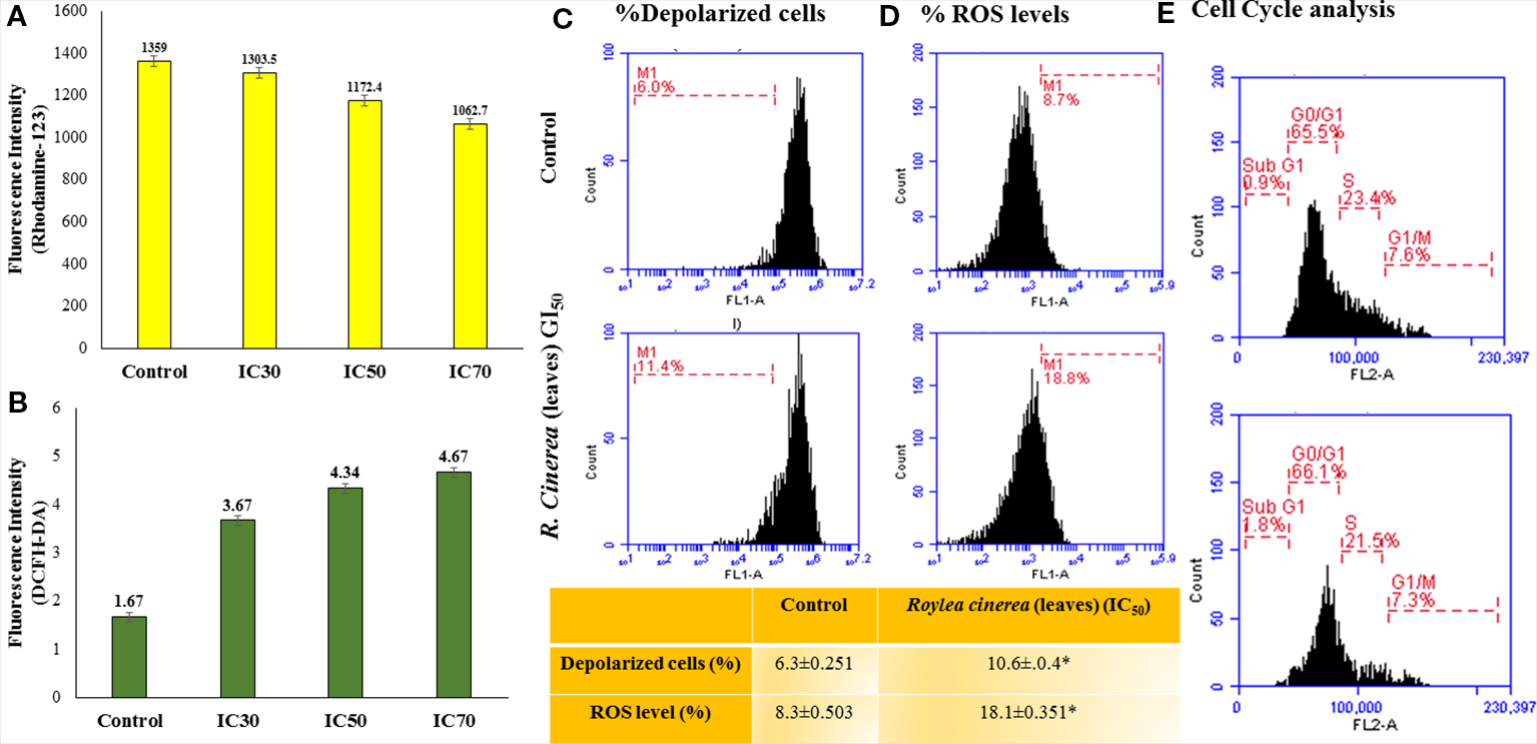
**(A)** MMP status assessed by Rhodamine-123 staining for leaves extract of *R. cinerea* in L6 cell line analyzed with Biotek multi-well plate reader for fluorescent intensity (485 nm excitation and 528 nm emission). **(B)** Reactive oxygen species (ROS) status assessed by DCFH-DA staining for leaf extract of *R. cinerea* analyzed with Biotek multi-well plate reader for fluorescent intensity (485 nm excitation and 528 nm emission). **(C)** MMP status assessed by Rhodamine-123 staining for leaves extract of *R. cinerea* in L6 cell line analyzed with BD Accuri TM C6 Flow Cytometer (excitation 488 nm, emission 535 nm, FL-1 channel, events recorded 10,000 per sample). *Difference between % depolarized cells in control cells and treated cells (IC_50_ *R. cinerea* (leaves) extract) statistically significant (Independent Student's t-test, p ≤ 0.5) **(D)** ROS status assessed by DCFH-DA staining for leaf extract of *R. cinerea* analyzed with BD AccuriTM C6 Flow Cytometer (excitation 511 nm, emission 535 nm, FL-1 channel, events recorded 10,000 per sample), *Difference between % ROS in control cells and treated cells (IC_50_ *R. cinerea* (leaves) extract) statistically significant (Independent Student's t-test, p ≤ 0.5) **(E)** Effect of methanolic leaves extract of *R. cinerea* on cell cycle analysis compared with non-treated cells (control).

Furthermore, the L6 cells were treated with the GI_50_ of leaves extract of *R. cinerea* for 24 h revealed significantly enhanced levels of intracellular ROS (**Figures 4B, D**). The elevated levels of intracellular ROS in L6 cells demonstrated the apoptogenic efficiencies of both the extracts. ROS is generated continuously in the body as a consequence of mitochondrial bioenergetics mainly oxidative metabolism. But these radicals (O_2_^.-^ superoxide anion, OH^.^ hydroxyl radical, OOH^.^ peroxide radical and H_2_O_2,_ etc.) are balanced *via* an indigenous cellular system. These radicals form an integral part of a network of cellular signaling pathways including cell proliferation and programmed cell death (Hansen et al., 2006; Menon and Goswami, 2007). However, imbalanced intracellular redox can target various biomarkers involved in cancer pathophysiology which includes CDK's (cyclin-dependent kinases), various transcriptional factors (Nrf2, FOXO3) and pro-apoptotic markers including MAPK's (Mates et al., 2012; Roleira et al., 2015). Phenolic compounds are known to show pleiotropic effects by acting as pro-oxidants in order to preserve normal cell cycle regulation *via* CDK's functions, suppress inflammation, tumor invasion combined with induction of apoptosis (Ziech et al., 2012). The active constituents present in *R. cinerea*, in spite of showing considerable *in vitro* antioxidant potential, might be stimulated to act as pro-oxidant in the state of imbalanced redox environment in L6 cells. Elevated levels of ROS also affects cell membrane, mitochondria, DNA, lipids, and proteins. Mitochondria play a crucial role in the process of induction of apoptosis as it contains various pro-apoptotic markers such as apoptotic proteases and cytochrome c. ROS can cause the opening of mitochondrial permeability transition pores and disruption of the electron transport chain which ultimately leads to apoptosis or cell death. The methanolic leaves extract (GI_50_) also substantially altered the mitochondrial membrane potential which ultimately leads to the opening of mitochondrial pores followed by the release of pro-apoptotic markers that lead to cell death (Bortner and Cidlowski, 1999) (**Figures 4A, C**).

**Figure 5:**
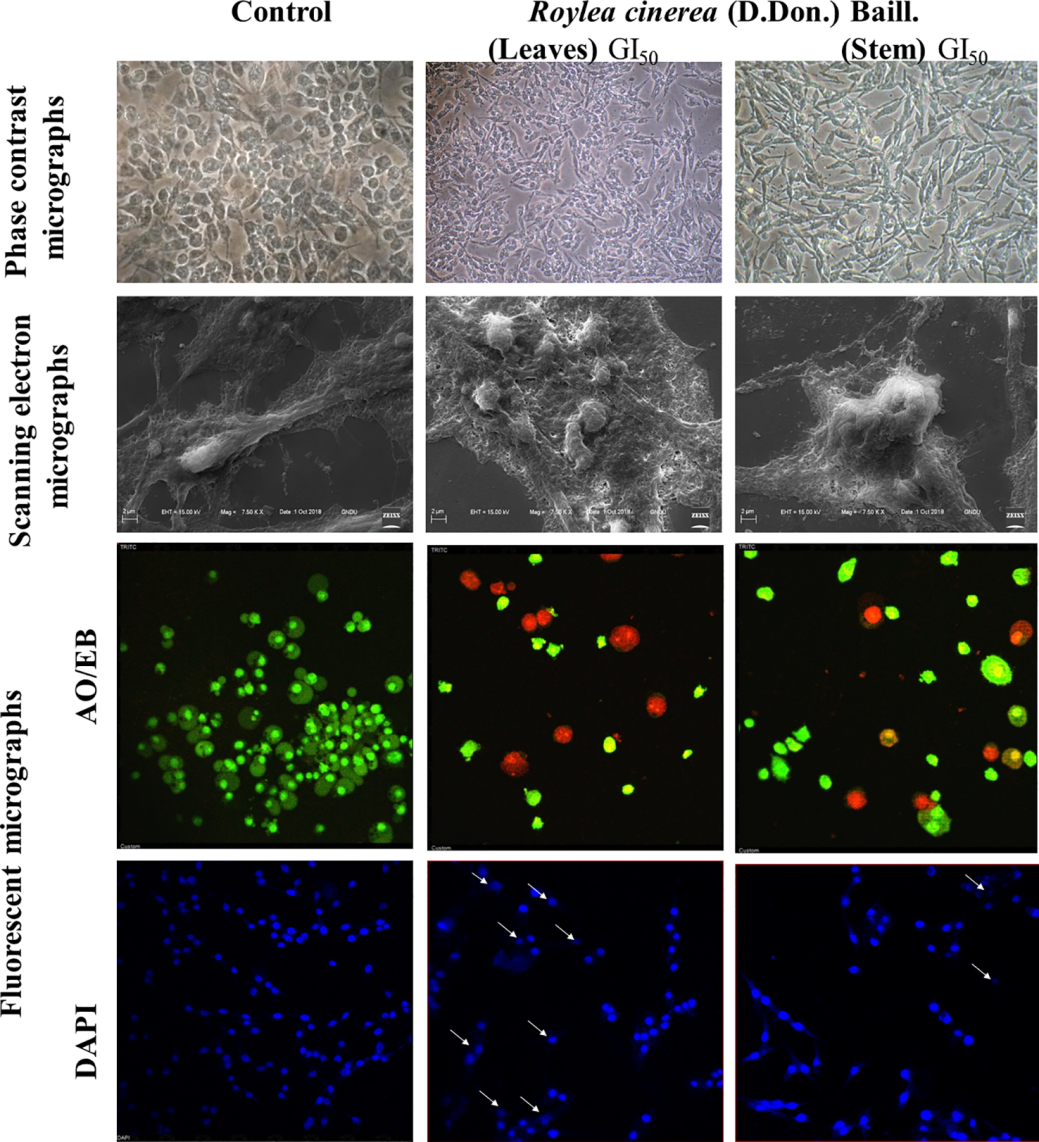
L6 cells imaged by phase contrast for morphological changes (40x); Scanning electron microscopy for surface variations (7.50 KX); Confocal microscopy for nuclear with DAPI and AO/EB (40x).

Mostly the anticancer drugs induce apoptosis through nuclease mediated destruction of DNA content in cells which leads to induction of cell cycle arrest. Thus, to elucidate the effects of methanolic leaves extract of *R. cinerea* on DNA content in L6 cells, cell cycle assay was performed using propidium iodide fluorescent dye. The membrane permeable PI dye intercalates with bases of DNA and represents the DNA content present in cells. In our experiment, the methanolic leaves extract of *R. cinerea* at GI_50_ concentration, showed the enhanced percentage of Sub-G1 phase from 0.9% to 1.8% which represents the apoptotic population when compared to control non treated cells. Further, cell population in G0/G1 phase was increased in treated cells from 65.5% to 66.1% with a decrease in S phase (23.4% to 21.5%) and G1/M phase (7.6% to 7.3%). The results indicated the increase in apoptotic cells and cell cycle arrest at G0/G1 phase in treated cells as compared to the non-treated cells which may be attributed to DNA damage mediated p53 activation to check further cell proliferation (**Figure 4E**) (Khan et al., 2019). The anticancer drugs specifically act by initiating various signaling pathways and ultimately inducing apoptosis. The mode of death induced by methanolic extract of leaves and stem of *R. cinerea* was confirmed through various *in vitro* experiments. Phase-contrast microscopy clearly depicted the presence of membrane blebbing, cell shrinkage, and apoptotic bodies in treated cells as compared to normal healthy cells in control. Confocal microscopy revealed various apoptotic features such as condensed nuclear material, flattened cytoplasmic borders, degradation of DNA into scattered masses in treated cells as compared to control L6 cells (**Figure 5**). AO/EB staining confirmed the presence of apoptotic cells (dark orange) in treated groups and live cells in control (green) (**Figure 5**). Furthermore, scanning electron microscopy studies clearly showed cell size reduction, blebbing of the membrane, rounding of cells, and apoptotic bodies. Thus, the results of the present study clearly corroborated with the association of phytochemicals (phenolics) in combating cancer *via.* pleiotropic effects and altering signaling pathways at the mitochondrial level.

**Figure 6:**
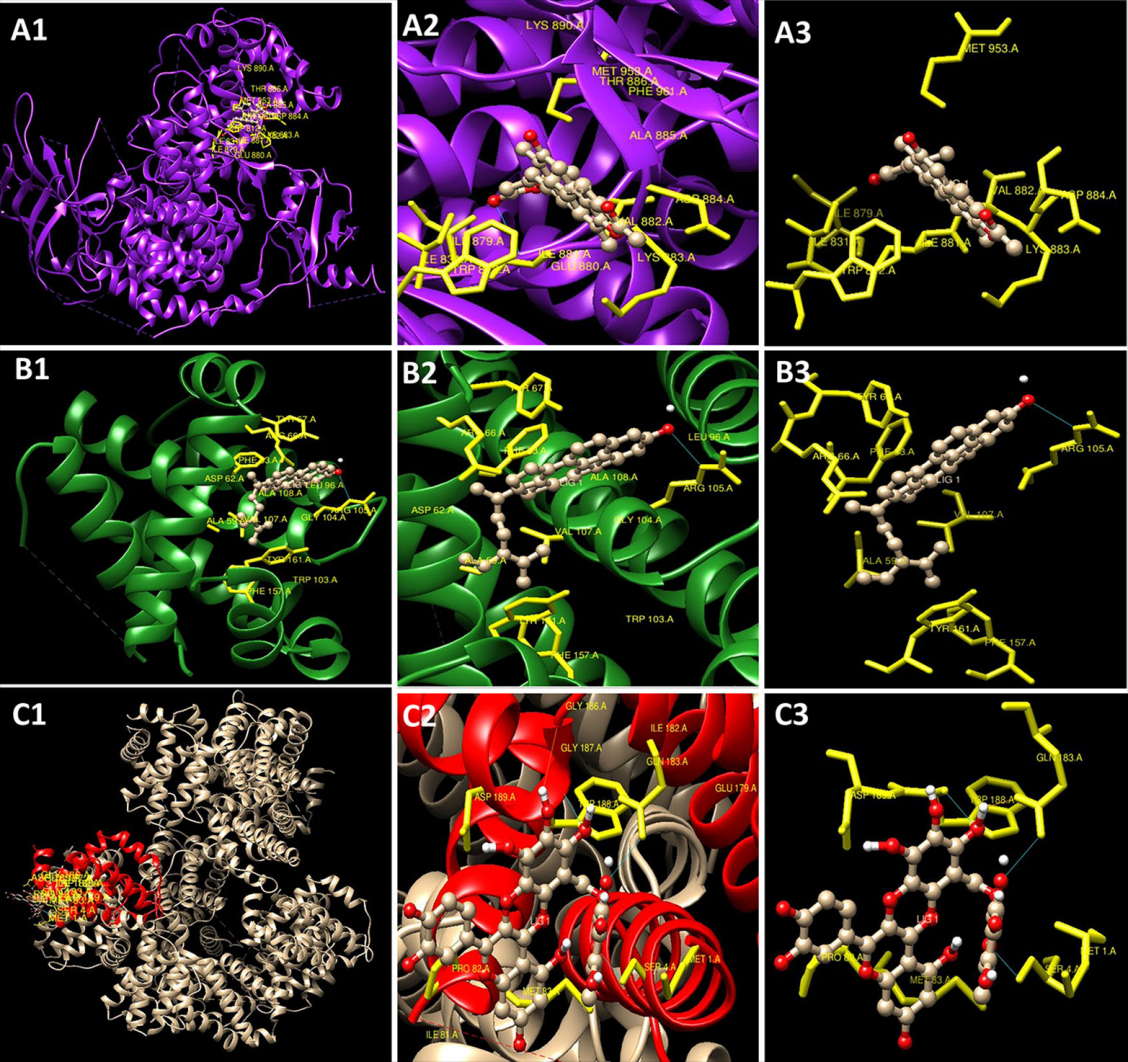
Docking conformations (Chimaera software) showing interaction of compounds with minimum binding energy **(A1**–**A3)**. Cinereanoid D with PI3K (PDB ID: 1E8Z) **(B1–B3)** Stigmasterol with Bcl2 (PDB ID: 4IEH) **(C1**–**C3)** Rutin with Bclxl (PDB ID: 4QNQ).

**Figure 7:**
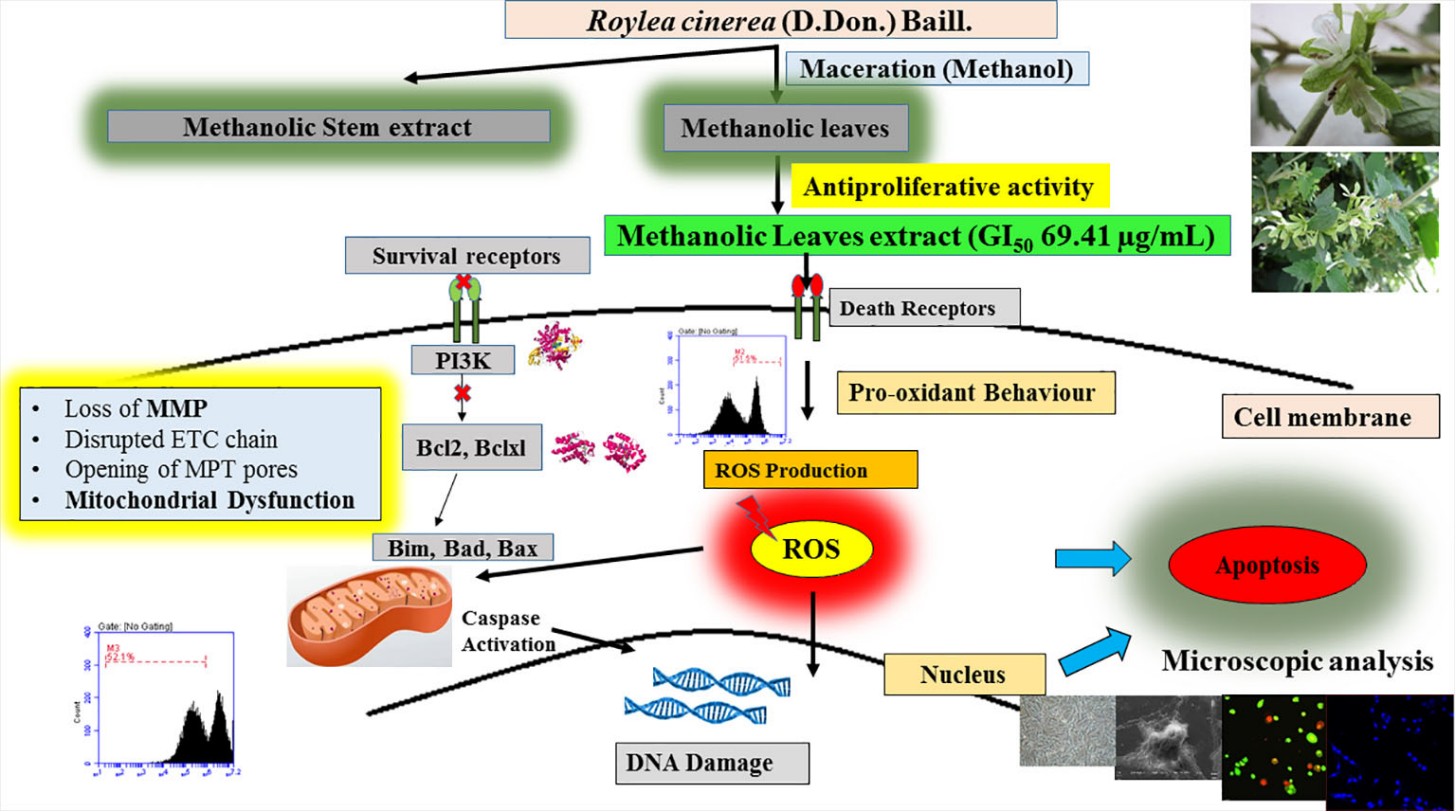
Detailed proposed mechanism involved in the induction of apoptosis in L6 cells by *R.cinerea* leaves extract.

The experimental results obtained paved the way to clarify the mechanism involved by which the leaves extract was able to activate caspase-3 activity in L6 cells. To obtain further information, docking studies were performed. Previously reported phyto-constituent present in *R. cinerea viz.,* 1-methyl-1-H-indole-3-carbaldehyde, β-lactam, β-sitosterol, calyone, cinereanoid A, cinereanoid B, cinereanoid C, cinereanoid D, pilloin, rutin, and stigmasterol were made to dock to the protein structure of PI3K (PDB ID: 1E8Z), Bcl2 (PDB ID: 4IEH), and Bclxl (PDB ID: 4QNQ). The docking pose with minimum binding energy was considered for further analysis through chimera software to get a clear picture of the ligand regarding its orientation, H-bonding, identification of residues and mode of interactions. It revealed that phytoconstituents present in the methanolic extract of leaves possessed a good binding affinity toward the protein targets (PI3K, Bcl2, and Bclxl). Docking confirmations were analyzed for each ligand which explicated interactions of different amino residues of protein targets with user-defined ligands through H-bond formation. Among various ligands, cinereanoid D showed minimum binding energy, i.e., -11.56 Kcal/mol and fits well in the binding cavity of PI3K protein target (1E8Z) (**Figures 6A1–A3**). However, for protein target 1E8Z, stigmasterol also showed H-bonding with minimum binding energy of -10.85 Kcal/mol (ASP 884 with bond length 3.016 Å) followed by calyone with binding energy -10.65 Kcal/mol (GLU 880 with bond length 2.730 Å), rutin with binding energy -10.10 Kcal/mol (GLU 814 and ALA 885 with bond length 2.881 Å and 2.835 Å), Cinereanoid C, Cinereanoid A (GLU 880 and ALA 885 with bond length 2.621 and 2.653 Å), β-sitosterol, Cinereanoid B (VAL 882 and LYS 883 with bond length 3.009 and 2.870 Å), pilloin (ALA 885 with bond length 2.899 Å) showed binding energy -10.00, -9.37, -9.16, -9.03, and -8.31 Kcal/mol, respectively (**Supplementary Figure 3**, **Table 1**). The PI3K pathway is one of the major pathway for cell growth and survival. Over-activation *via* PDGFR and EGFR families (oncogenic targets) of this preordained PI3K pathway can lead to frequent incidences of cancer. This assumption makes it an obvious target for cancer treatment *via* developing some promising isoform specific PI3K inhibitors such as class Ia PI3-kinases. Class Ia PI3 kinases have been well documented for transmitting signals for survival responses through PKB/AKT activation. ATP binding sites/pocket as a catalytic domain present on PI3Kinases can occur as a probable target of PI3K inhibitors to bind. Labdane diterpenoids are commonly found in family Lamiaceae. In cinereanoid D, butenolide side chain (furan) is present with a hydroxyl group at 16-C. Literature studies showed scarce scientific evidence regarding the structure activity relationship of novel labdane diterpenoids cinereanoid A-D. Labdane diterpenoids are reported to affect DNA synthesis facilitated by the presence of a double bond at its C7-C8 position (Dimas et al., 1998). However, stigmasterol have been well reported for its efficacy to inhibit cancer development and progression in both *in vitro* and *in vivo* system (Zhang et al., 2016). PI3 Kinases through AKT activates Bcl2 family proteins. Protein targets Bcl2 and Bclxl are basically anti-apoptotic/pro-survival proteins with four conserved domains BH1, BH2, BH3, and BH4. In response to death signals, the BH3 domain is neutralized by pro-apoptotic proteins, i.e., Bad, Bmf which leads to the release of cytochrome c for apoptosis through disturbing the integrity of the mitochondrial membrane. Overexpression of Bcl2 family proteins may be responsible for the progression of cancer. For the protein target Bcl2 (4IEH), among selected ligands, stigmasterol showed minimum binding energy of -9.81 Kcal/mol (ARG 105 with bond length 3.079 Å) to binding cavity (**Figures 6B1–B3**) followed by cinereanoid A, rutin, calyone, β-sitosterol, cinereanoid D, cinereanoid C, cinereanoid B, pilloin with minimum binding energy -9.48, -9.09 (GLY 104 with bond length 2.772 Å), -8.77 (ARG 105 and TYR 67 with bond length 3.023 and 2.998 Å), -8.61 (TYR 161 with bond length 2.859 Å), -8.09, -8.05, -7.50, and -7.08 Kcal/mol (ARG 66 with bond length 3.097 Å), respectively.1-methyl-1-H-indole-3-carbaldehyde and β-lactam showed minimum binding energy -4.74 and -4.15 Kcal/mol (**Supplementary Figure 4**, **Table 1**). Furthermore, for the protein target Bclxl (4QNQ), maximum affinity to the binding cavity was shown by rutin -9.31 Kcal/mol with H-bond formation with amino acid residues GLN 183 (3.013 Å), TRP 188 (3.001 Å), SER 4 (3.005 Å) (**Figures 6C1–6C3**) followed by cinereanoid D, cinereanoid A, cinereanoid B, calyone, β-sitosterol, cinereanoid C, stigmasterol, pilloin, 1-methyl-1-H-indole-3-carbaldehyde and β-lactam with minimum binding energy -8.38, -8.36 (ALA 93 with bond length 2.660 Å), -8.14 (ALA 93 with bond length 2.512 Å), -8.08, -8.01, -7.78, -7.43, -7.28, -4.71, and -4.39, respectively. The ligands which did not show any H-bonding were still well fit into the binding cavity of the protein which may be attributed to electrostatic, Van der waal forces and hydrophobic interactions (**Supplementary Figure 5**, **Table 1**). Thus, the experimental findings confirming loss of mitochondrial membrane potential, generation of ROS and cell cycle arrest at G0/G1 phase by the methanolic extract of leaves of *R*.*cinerea* corroborated with the docking studies showing the synergistic potential of its phytoconstituents in the induction of apoptosis in immortalized L6 skeletal muscle cell line (**Figure 7**).

**Table 1:** Predicted binding energies for constituents present in *Roylea cinerea* (D.Don) Baill. docked with PI3K (PDB ID: 1E8Z), Bcl2 (PDB ID: 41EH), Bclxl (PDB ID: 4QNQ) obtained from www.rcsb.org.

S.No.	Molecules	PI3K (PDB:1E8Z)		Bcl2 (PDB:4IEH)		Bclxl (PDB ID: 4QNQ)	
		Minimum binding energy (Kcal/mol)	No. of H-bonds	Minimum binding energy (Kcal/mol)	No. of H-bonds	Minimum binding energy (Kcal/mol)	No. of H-bonds
**1.**	1-methyl-1-H-indole-3-carbaldehyde	-5.38		-4.74	0	-4.71	0
**2.**	β-lactam	-5.47	1	-4.15	0	-4.39	0
**3.**	β-sitosterol	-9.16	1	-8.61	1	-8.01	0
**4.**	Calyone	-10.65	0	-8.77	2	-8.08	0
**5.**	Cinereanoid A	-9.37	2	-9.48	0	-8.36	0
**6.**	Cinereanoid B	-9.03	2	-7.50	0	-8.14	0
**7.**	Cinereanoid C	-10.00	0	-8.05	0	-7.78	1
**8.**	Cinereanoid D	**-11.56**	1	-8.09	0	-8.38	1
**9.**	Pilloin	-8.31	1	-7.08	1	-7.28	0
**10.**	Rutin	-10.10	2	-9.09	1	**-9.31**	3
**11.**	Stigmasterol	-10.85	1	**-9.81**	1	-7.43	0

The bold values represent compounds with minimum binding energy.

## Conclusion

The present study revealed the antioxidant potential and DNA protective abilities of methanolic extracts of leaves and stem of *R. cinerea* along with antiproliferative and apoptosis induction potential against immortalized L6 cell line. However, the methanolic leaves extract of *R. cinerea* showed better activity as compared to stem extract. Furthermore, mechanistic analysis revealed that methanolic extracts of leaves of *R. cinerea* induced apoptosis basically through increasing intracellular ROS generation, decreasing mitochondrial membrane potential and ultimately lead to cell death *via* apoptosis. Further, the experimental findings were strengthened by docking with already reported phytoconstituents of *Roylea cinerea* in literature with PI3 kinase and anti-apoptotic/pro-survival proteins. The study provided partial evidence for a pharmacological basis regarding clinical applications of *Roylea cinerea* in the treatment of cancer and will add significant information to establish a strong base to conduct further research on this plant and its unexplored health benefits. However, further *in vivo* experiments are required to confirm the efficacy and mechanism of action regarding this plant.

## Data Availability Statement

All datasets generated for this study are included in the article/**Supplementary Material**.

## Author Contributions

AB: formal analysis, investigation, methodology, data curation, and writing—original draft. HS and RA: writing, reviewing, and editing. AS and SK: intellectual contribution and reviewing manuscript. SA: conceptualization, supervision and project administration, reviewing and editing, and resources. BS: conceptualization, methodology, reviewing and editing, and supervision. All authors read and approved the final manuscript.

## Funding

The present study was supported by the University Grants Commission (UGC), New Delhi under the Rajiv Gandhi National Fellowship scheme to AB (vide grant no. 201415-RGNF-2014-15-SC-PUN-68052).

## Conflict of Interest

The authors declare that the research was conducted in the absence of any commercial or financial relationships that could be construed as a potential conflict of interest.

The handling editor declared a past co-authorship with one of the authors HS.

## Supplementary Material

Supplementary Figure 1
**** Structure of phyto-constituents (Ligands) present in *R. cinerea* prepared using Chemsketch software for docking analysis.

Supplementary Figure 2
**** Structure of protein PI3K (1E8Z), Bcl2 (4IEH), Bclxl (4QNQ) and their binding sites obtained from www.rcsb.org.

Supplementary Figure 3 Docking conformations of the phytoconstituents present in *R. cinerea viz*., a) 1-methyl-1-H-indole-3-carbaldehyde b) β-lactam c) β-sitosterol d) calyone e) cinereanoid A f) cinereanoid B g) cinereanoid C h) cinereanoid D i) pilloin j) rutin, and k) stigmasterol with PI3K (1E8Z).

Supplementary Figure 4 Docking conformations of the phytoconstituents present in *R. cinerea viz*., a) 1-methyl-1-H-indole-3-carbaldehyde b) β-lactam c) β-sitosterol d) calyone e) cinereanoid A f) cinereanoid B g) cinereanoid C h) cinereanoid D i) pilloin j) rutin, and k) stigmasterol with Bcl2 (4IEH).

Supplementary Figure 5 Docking conformations of the phytoconstituents present in *R. cinerea viz*., a) 1-methyl-1-H-indole-3-carbaldehyde b) β-lactam c) β-sitosterol d) calyone e) cinereanoid A f) cinereanoid B g) cinereanoid C h) cinereanoid D i) pilloin j) rutin, and k) stigmasterol with Bclxl (4QNQ).

Supplementary Figure 6 Standard curve for ascorbic acid (20–200 µg/ml) for the calculation of electron donating capacity (molybdate ion reduction assay).
