# Supplementary figures and images for: Antiproliferative Effects of Roylea cinerea (D. Don) Baillon Leaves in Immortalized L6 Rat Skeletal Muscle Cell Line: Role of Reactive Oxygen Species Mediated Pathway
Source: Front Pharmacol. 2020 Mar 13;11:322. doi: 10.3389/fphar.2020.00322 (PMC7083017; doi:10.3389/fphar.2020.00322)

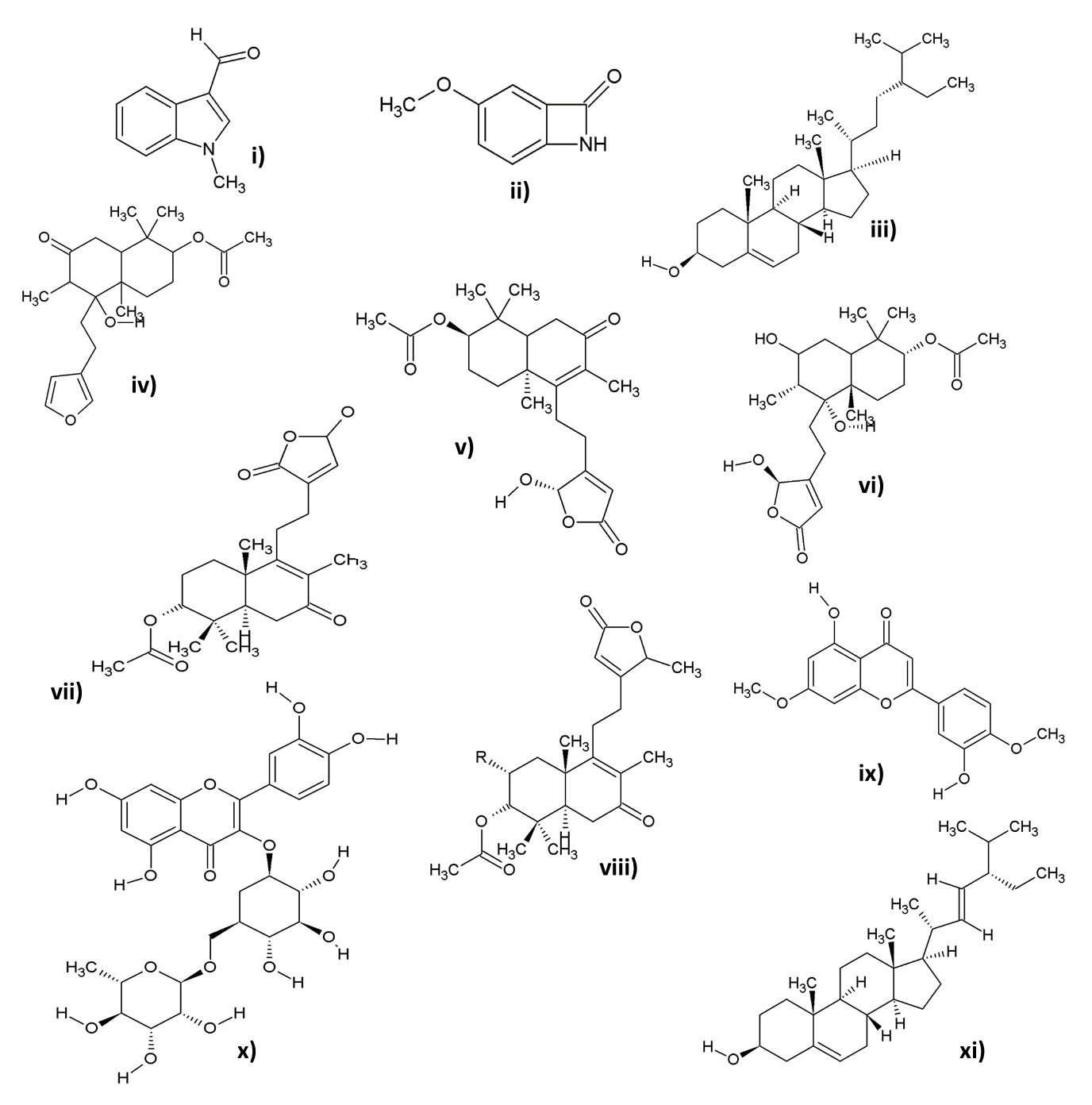

Supplement: Supplementary Figure 1 — Structure of phyto-constituents (Ligands) present in R. cinerea prepared using Chemsketch software for docking analysis. [file Image_1.tif]

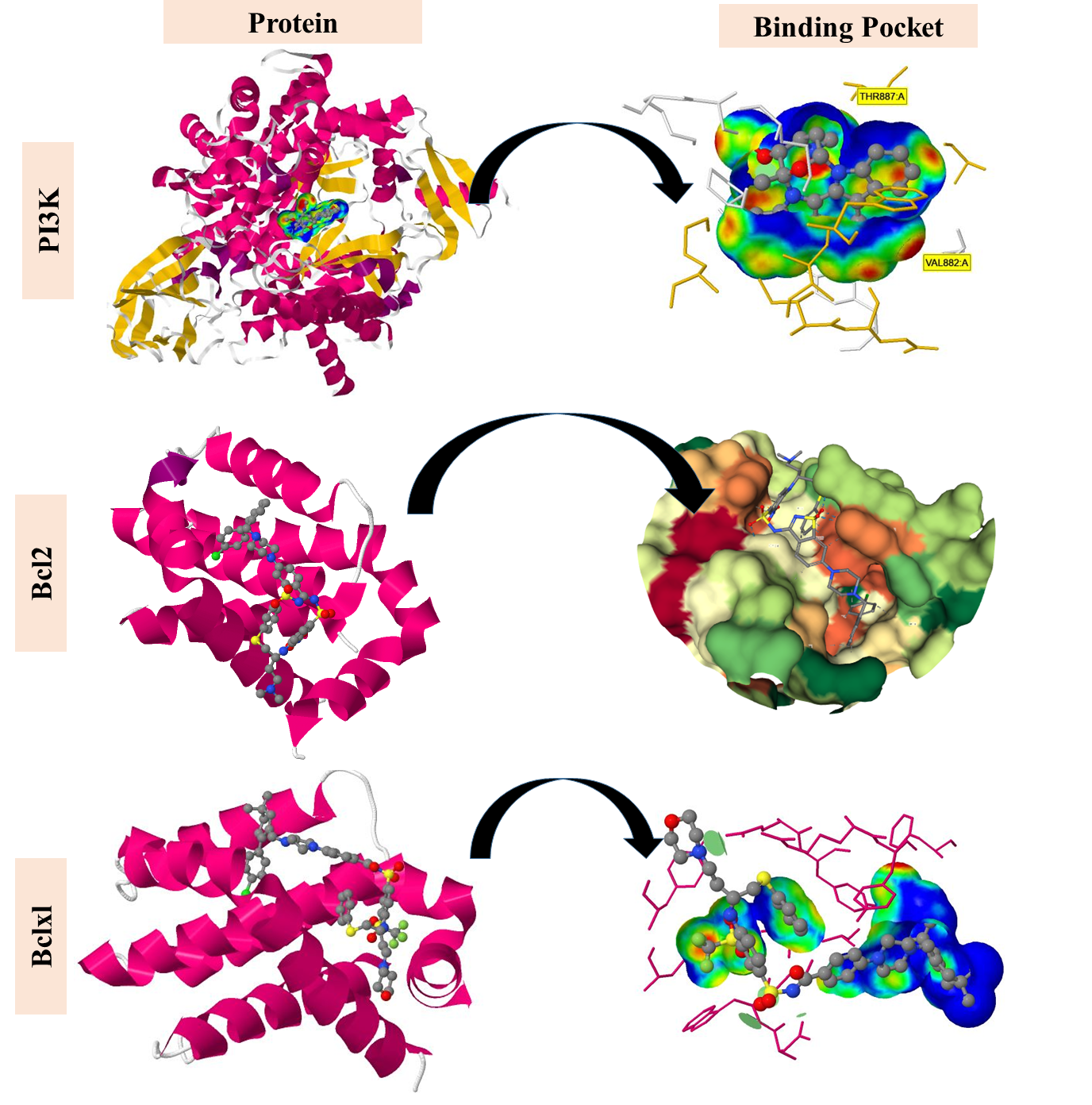

Supplement: Supplementary Figure 2 — Structure of protein PI3K (1E8Z), Bcl2 (4IEH), Bclxl (4QNQ) and their binding sites obtained from www.rcsb.org. [file Image_2.tif]

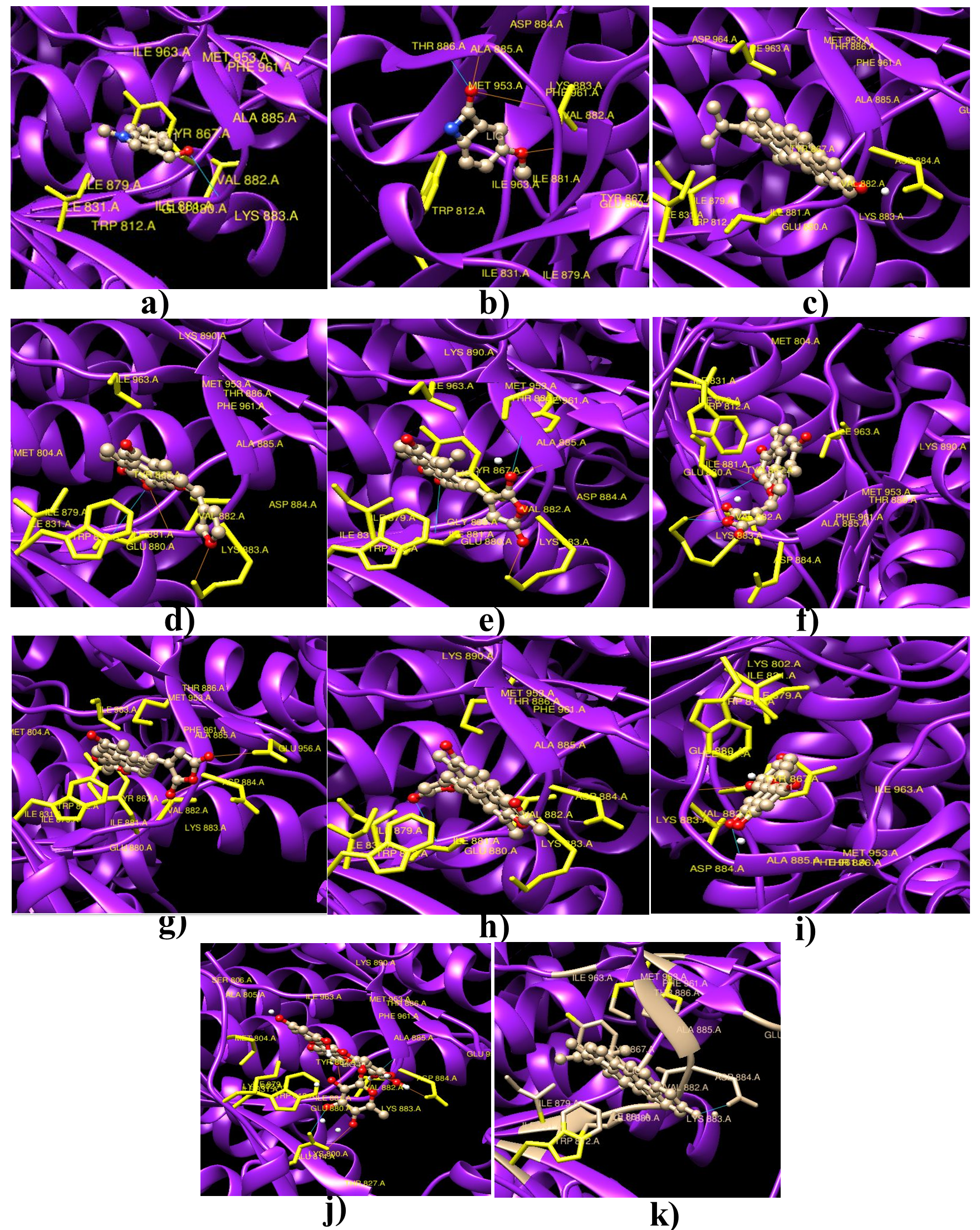

Supplement: Supplementary Figure 3 — Docking conformations of the phytoconstituents present in R. cinerea viz., a) 1-methyl-1-H-indole-3-carbaldehyde b) β-lactam c) β-sitosterol d) calyone e) cinereanoid A f) cinereanoid B g) cinereanoid C h) cinereanoid D i) pilloin j) rutin, and k) stigmasterol with PI3K (1E8Z). [file Image_3.tif]

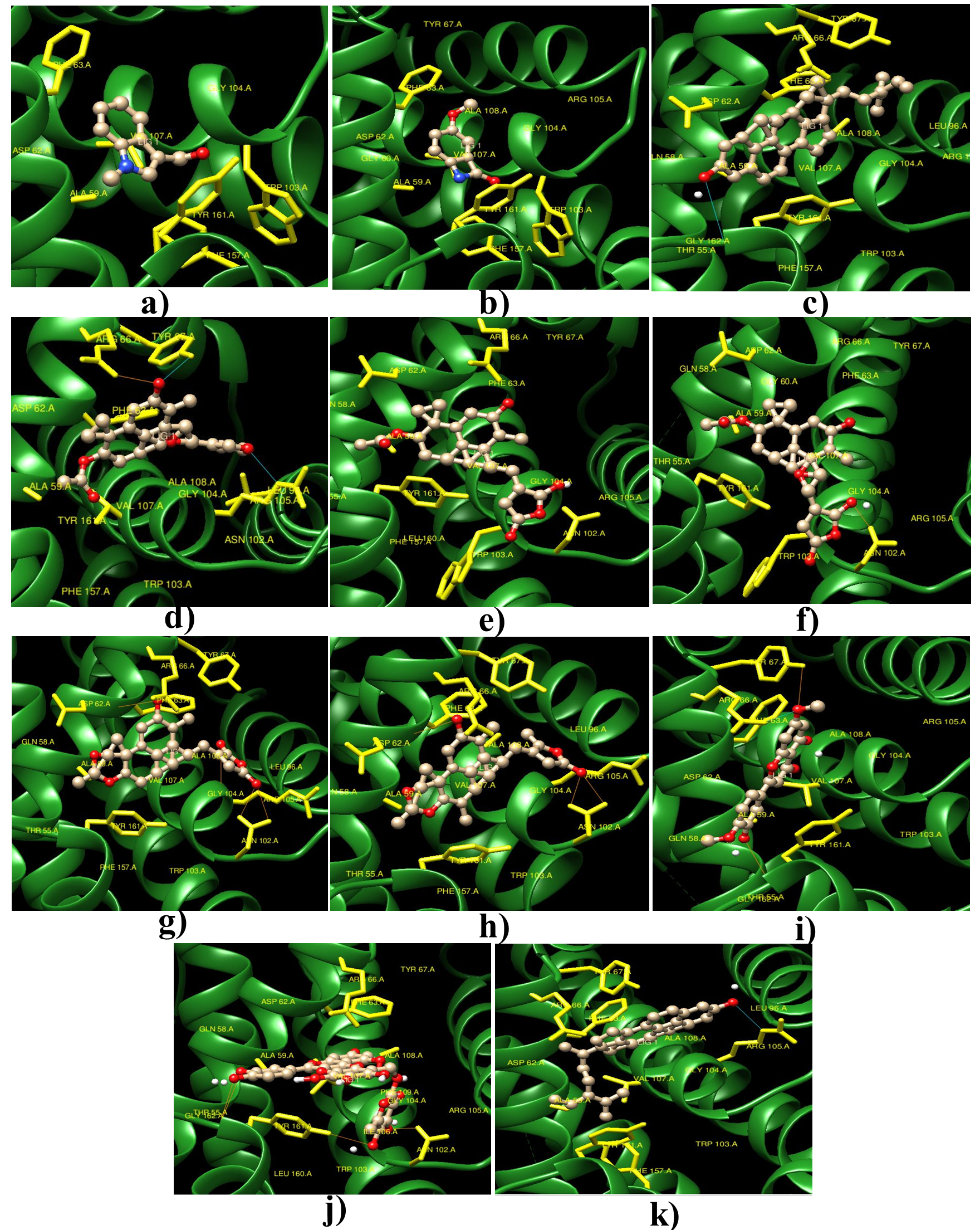

Supplement: Supplementary Figure 4 — Docking conformations of the phytoconstituents present in R. cinerea viz., a) 1-methyl-1-H-indole-3-carbaldehyde b) β-lactam c) β-sitosterol d) calyone e) cinereanoid A f) cinereanoid B g) cinereanoid C h) cinereanoid D i) pilloin j) rutin, and k) stigmasterol with Bcl2 (4IEH). [file Image_4.tif]

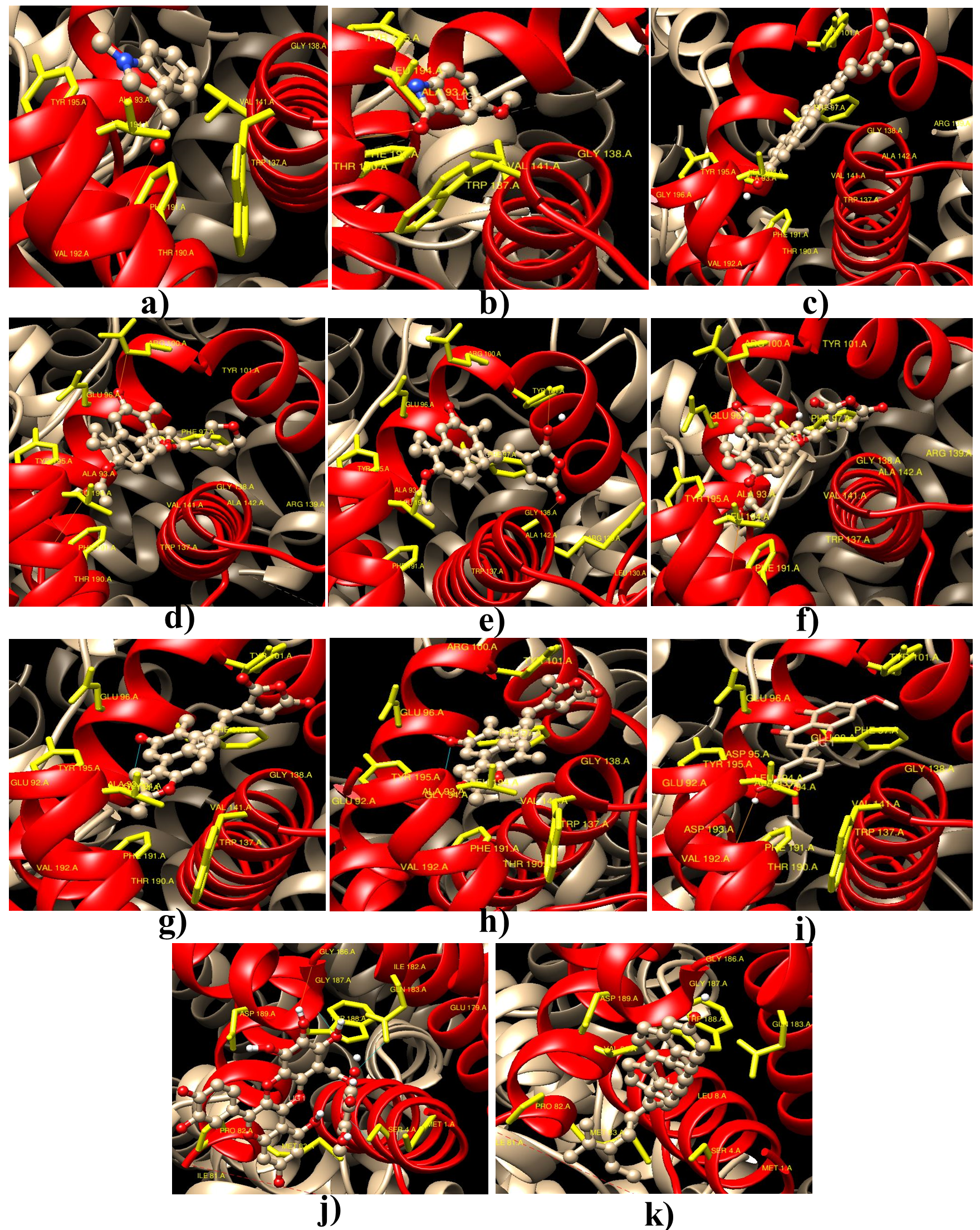

Supplement: Supplementary Figure 5 — Docking conformations of the phytoconstituents present in R. cinerea viz., a) 1-methyl-1-H-indole-3-carbaldehyde b) β-lactam c) β-sitosterol d) calyone e) cinereanoid A f) cinereanoid B g) cinereanoid C h) cinereanoid D i) pilloin j) rutin, and k) stigmasterol with Bclxl (4QNQ). [file Image_5.tif]

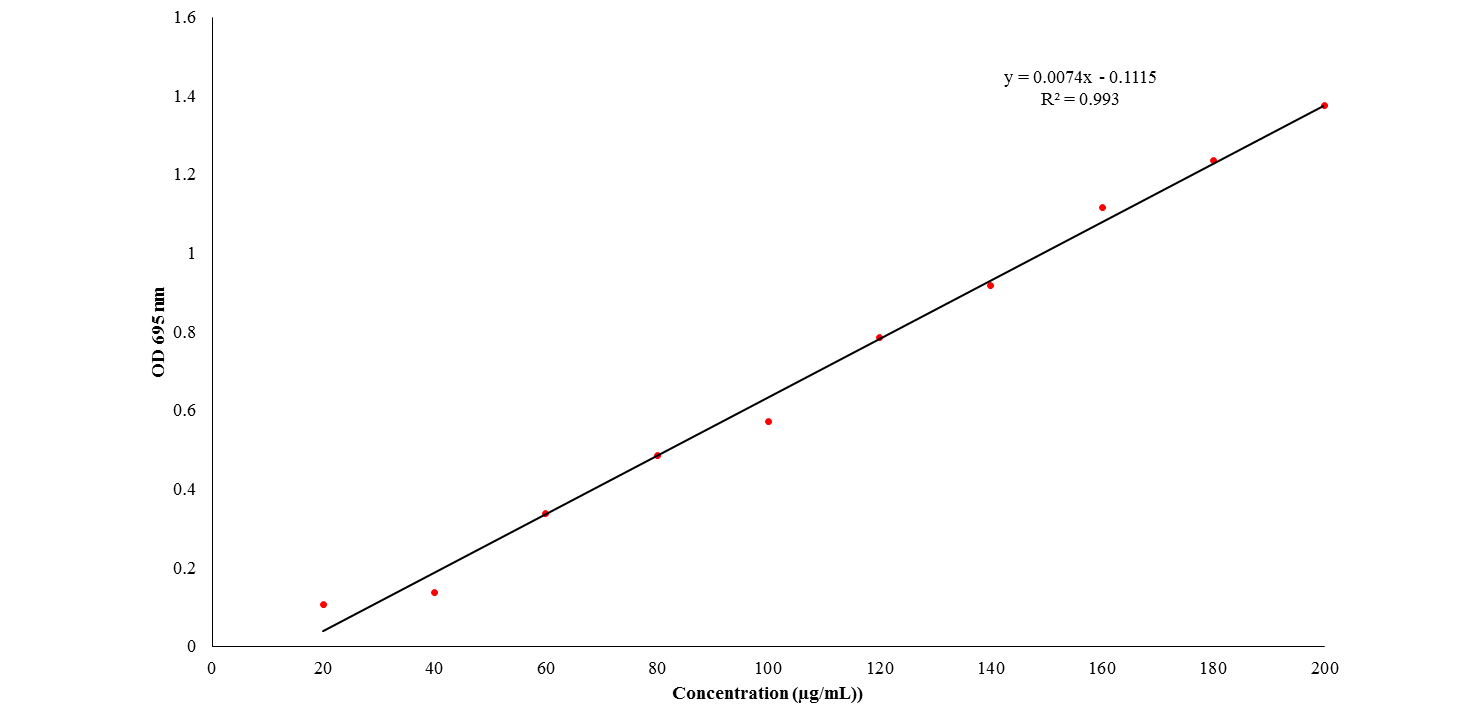

Supplement: Supplementary Figure 6 — Standard curve for ascorbic acid (20–200 µg/ml) for the calculation of electron donating capacity (molybdate ion reduction assay). [file Image_6.tif]
